# The association of body mass index with difficult tracheal intubation management by direct laryngoscopy: a meta-analysis

**DOI:** 10.1186/s12871-018-0534-4

**Published:** 2018-06-30

**Authors:** Tingting Wang, Shen Sun, Shaoqiang Huang

**Affiliations:** Department of Anaesthesia, Obstetrics & Gynecology Hospital, Fudan University, 128# Shenyang road, Shanghai, 200090 China

**Keywords:** Intubation, Intratracheal, Body mass index, Meta-analysis

## Abstract

**Background:**

Obesity is a serious disorder and may bring about many difficulties of perioperative management. A systematic review was conducted to assess the association between obesity and difficult intubation.

**Methods:**

We searched electronic databases for related reviews and references of meta-analyses on August 14, 2017. The databases of PubMed, Embase, and the Cochrane controlled trials register were searched compared obese with non-obese patients in which difficult intubation rate of the adult population were retrieved. Patients with a BMI ≥ 30 kg·m^− 2^ were considered obese. The primary outcome was difficult tracheal intubation; secondary outcomes were the rates of difficult laryngoscopy and Mallampati score ≥ 3. This review included papers published from 1998 to 2015.

**Results:**

This review included 204,303 participants in 16 studies. There was a statistically significant association between obesity and risk of difficult tracheal intubation (pooled RR = 2.04, 95% CI: 1.16–3.59, *p =* 0.01; I^2^ = 71%, *p =* 0.008, Power = 1.0). It also showed significantly association between obesity and risk of difficult laryngoscopy (pooled RR = 1.54, 95% CI: 1.25–1.89, *p < 0.0001*; I^2^ = 45%, *p =* 0.07, Power = 1.0), obesity and risk of Mallampati score ≥ 3 (pooled RR = 1.83, 95% CI: 1.24–2.69, *p =* 0.002; I^2^ = 81%, *p <* 0.00001, Power = 0.93). However, there were no association of obesity and risks of difficult intubation compared with non-obesity in the cohort studies (pooled RR = 3.41, 95% CI: 0.88–13.23, *p = 0.08*; I^2^ = 50%, *p =* 0.14) and the elective tracheal intubation (pooled RR = 2.31, 95% CI: 0.76–6.99, *p = 0.14*; I^2^ = 73%, *p =* 0.01), no associated with an increased risk of difficult laryngoscopy in the sniffing position (pooled RR = 2.00, 95% CI: 0.97–4.15, *p = 0.06*; I^2^ = 67%, *p =* 0.03).

**Conclusion:**

Obesity was associated with an increased risk of difficult intubation, difficult laryngoscopy and Mallampati score ≥ 3 in adults patients undergoing general surgical procedures. However, there were no association of obesity and risks of difficult intubation compared with non-obesity in the cohort studies and the elective tracheal intubation, no associated with an increased risk of difficult laryngoscopy in the sniffing position. Future analyses should explore the association of BMI and difficult airway.

## Background

Obesity is a public health issue that leads to serious social, psychological and physical problems [1]. According to the World Health Organization survey, obesity rates have almost doubled worldwide since 1980 [2]. With the growing number of obese adults, increasing attention is being paid to difficult intubation (DI).

Although several tools (such as video laryngoscope, fibre-optic tracheal airway devices) can facilitate intubation or increase success rates, a DI can still be challenging for anaesthetists. The higher cost and uncomfortable nature of awake intubation compared with traditional laryngoscopy are common contributing causes to the difficulty [3, 4]. Accordingly, a direct laryngoscope (DL) remains the most widely used device for tracheal intubation [5].

Furthermore, there is no consensus about whether obesity is associated with the occurrence of a DI. For instance, Shiga et al. found the rate of DI in obese patients (body mass index > 30) to be more than three times that in normal patients [6]. Conversely, some studies after 2005 reached a different conclusion, indicating that a high body mass index (BMI) was not associated with DI [7, 8]. In addition, although most anaesthesiologists recommend the sniffing position and consider it to be essential for improving tracheal intubation [9], the superiority of this position has been questioned during the last decade [10].

Considering the points raised above, we performed a review to evaluate the association between BMI and DI using meta-analysis and furthermore stratified by study design (cohort or case-control) and position (sniffing or supine). The primary outcome was to compare the rate of difficult tracheal intubation in high BMI vs. low BMI patients with a DL. The secondary outcomes were the rate of difficult laryngoscopy and a Mallampati score ≥ 3.

## Methods

### Search strategy

This systematic review was performed in accordance with the Preferred Reporting Items for Systematic Reviews and Meta-analyses (PRISMA) guidelines.

The protocol was registered with PROSPERO under number CRD42017058340 on August 14, 2017.

We searched electronic databases for related reviews and references of meta-analyses on August 14, 2017. To identify relevant articles, searches in PubMed, Embase, and the Cochrane controlled trials register (CENTRAL) were performed using the keywords “failed tracheal intubation”, “difficult tracheal intubation”, “difficult laryngoscopy”, “Cormack Lehane” , “Mallampati”, “BMI” and “obesity” as MeSH components and text words. There were no limitations regarding language, time of publication or article type. To reduce publication bias, ongoing studies at ClinicalTrials.gov and proceedings from the American Society of Anesthesiologists (ASA) annual meetings over the last 5 years (from July 2012 to August 2017) were also retrieved.

### Inclusion and exclusion criteria

Studies meeting the following criteria were included: (1) reference to humans; (2) DI rate as an outcome using an adult population; (3) comparison of obese with non-obese patients according to BMI and (4) results reported or obtained via calculation of effect estimates of the relative risk (RR), hazard ratio (HR) or odds ratio (OR) with 95% confidence intervals (CIs). BMI was calculated by dividing the patient’s body weight in kilograms by the square of their height in metres. Patients with a BMI ≥ 30 kg·m^− 2^ were considered obese. All types of surgery were considered.

The exclusion criteria were as follows: (1) case reports, cross-section studies, editorials, reviews and abstracts; (2) known risk factors for difficult airway (traumatic facial abnormalities, airway malformation and pathology, cervical spine fractures, those with a history of airway or intubation difficulty); and (3) pre-hospital tracheal intubation, that is, studies of pre-hospital tracheal intubation were excluded because the airway management setting differs between out-of-hospital and in-hospital. Accordingly, a non-planned endotracheal intubation in a hospital was defined as an emergency tracheal intubation.

### Primary and secondary outcomes

The primary outcome was rate of difficult tracheal intubation. The secondary outcomes were 1) rate of difficult laryngoscopy and 2) a Mallampati score ≥ 3. In 1993, the ASA has defined difficult endotracheal intubation as 3 attempts at endotracheal intubation when an average laryngoscope is used or when endotracheal intubation takes 10 min or more [11]. Then these Practice Guidelines were update in “Practice Guidelines for Management of the Difficult Airway” in 2013, and difficult tracheal intubation was defined as requiring multiple attempts in the presence or absence of tracheal pathology [12]. However, the concept was subjective and ambiguous. The Intubation Difficulty Scale (IDS) score, an objective scoring system that consists of numerical expressions of parameters and has been validated in many studies, was proposed to assess intubation difficulty in a standardized manner [13]. An IDS score of 0 means easy intubation, 1 to 5 means slight difficulty, and > 5 means moderate to major difficulty [14]. Thus, difficult intubation has been defined as requiring multiple attempts to place the tracheal tube into the trachea, lasting > 10 min using conventional laryngoscopy, or both, or and ISD > 5. Difficult laryngoscopy was assessed using the Cormack and Lehane Grades, classified into 4 grades: 1) visible vocal cords; 2) visible posterior commissure and epiglottis; 3) only epiglottis visible; and 4) no visible glottal structures. Grades 3 and 4 are considered a difficult laryngoscopy [15]. Mallampati scores were classified into 4 grades: 1) the tonsils, uvula and soft palate fully visible; 2) the soft palate and uvula visible; 3) the soft palate and base of uvula visible; and 4) only the hard palate visible [16]. Mallampati grades III or IV may be associated with difficult tracheal intubation.

### Data collection

Data were retrieved independently by two researchers (T. W. and S. S.); disagreements were considered by a third researcher (S. H.) and discussed until a consensus was reached. The discussion focused on whether the data conformed to the included criterion. One researcher (T. W.) designed a standard data form, and the other researchers (S. H. and S. S.) amended and validated the design before it was implemented. The authors of the retrieved studies were contacted (by S. H.) and asked to provide missing data that had not been reported or obtained by calculating the effect estimates of the RR, HR or OR with a 95% CI. If a response was not provided, the article was excluded. All of the studies were screened. The dataset included the name of the first author, year and country of publication, group situation (specified BMI to define obesity), number of participants, participant characteristics, study design and outcomes.

### Quality assessment

Risk of bias assessment was performed by two reviewers independently (S. H., T. W.) using the Risk of Bias in Non-randomized Studies – of Interventions (ROBINS-I) tool for observational studies [17]. The ROBINS-I tool assesses bias across six domains: confounding, participant selection, intervention classification, departure from intended interventions, missing data, measurement of outcomes and selection of reported results. For each domain, an outcome of low, moderate, serious, critical and no information for risk of bias is recorded. The overall risk of bias judgement is then determined through a combination of the six domains.

### Statistical analysis

Review Manager (RevMan version 5.2.5; The Nordic Cochrane Centre, The Cochrane Collaboration, Copenhagen, Denmark) was utilized for data analysis. As the outcome of the study was rare among all populations, ORs and HRs were directly considered as RRs in this study [18]. A *p* value ≤0.05 was considered statistically significant. The I^2^ statistic was utilized for heterogeneity assessment, and I^2^ > 50% was considered to indicate significant heterogeneity. A random-effects model was accepted for data analysis in the case of heterogeneity, and a fixed-effect model was adopted when heterogeneity was not found. Sources of heterogeneity were investigated by analysis of prespecified subgroups, as defined according to the study design (cohort or case-control) and position (sniffing or supine). The sniffing position was defined as patients with pillows or towels under their shoulders, with the head elevated and neck extended [19]. The supine position was defined as patients lying supine or not specifically in the sniffing position. To control the Type I error rate for multiple hypothesis testing, we used the Bonferroni correction as follows:

$$ {\upalpha}^{\ast}\kern0.5em =\kern0.5em \frac{\alpha }{c} $$

where α* is our new alpha level, α is our a priori significance level of 0.05 for the family of comparisons, and c is the number of comparisons [20]. We calculated the power for the primary outcome using post hoc power analysis with G*Power 3.1 software [21, 22]. Sensitivity analyses were conducted to assess the robustness of the data by removing each study sequentially and excluding those with emergency tracheal intubation, those defining obesity as a BMI cut-off > 30, and those in which parturients were participants. Potential publication bias was evaluated with a funnel plot; in the absence of bias, these plots resemble a symmetrical inverted funnel.

## Results

### Study selection

**Fig. 1:**
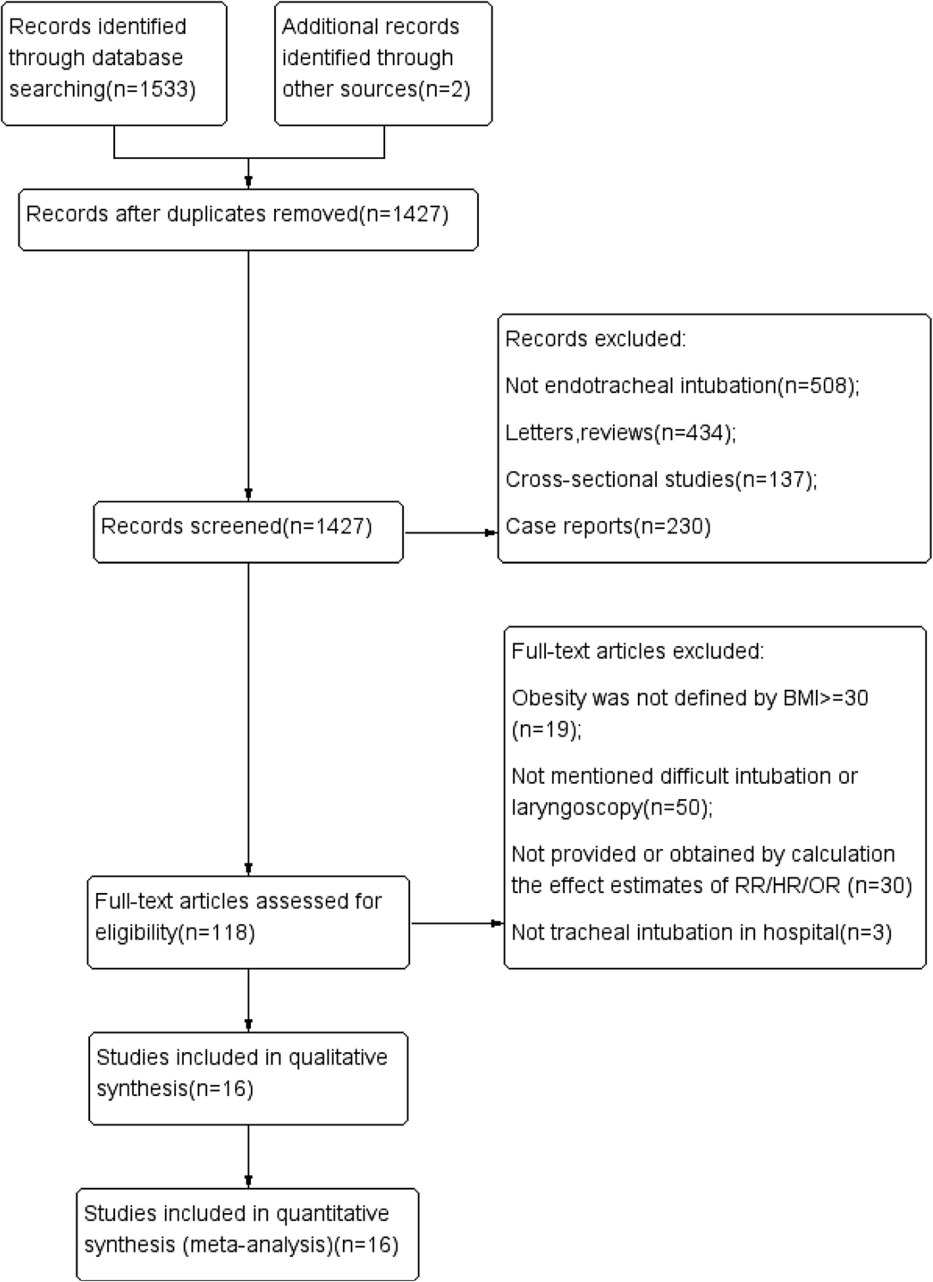
A total of 1533 related studies were obtained from the database search, and 2 citations were retrieved from the manual reference list search of the eligible studies. There were 108 studies that were removed because they were duplicates. Then, we excluded 968 studies after the initial review of the title and 341 after the abstract was reviewed due to they are not endotracheal intubation, letters, reviews, cross-sectional studies and case reports. There were 118 studies that were considered relevant and were read in full. Then 102 articles were excluded for reasons such as obesity was not defined as BMI = 30, not mentioned difficult intubation or laryngoscopy, not provided or obtained by calculation the effect estimates of RR/HR/OR, not tracheal intubation in hospital. After reviewing the full texts, 16 studies (published between 1998 and 2015) were selected for inclusion

A total of 1,533 related studies were obtained from the database search, and 2 citations were retrieved from a manual reference list search of the eligible studies. In total, 108 studies that were removed because they were duplicates. We then excluded 968 studies after the initial review of the title and 341 after the abstract was reviewed. Overall, 118 studies were considered relevant and were read in full. Then, 102 articles were excluded for reasons such as obesity was not defined as BMI ≥ 30, difficult intubation or laryngoscopy was not mentioned, the study did not provide or obtain the effect estimates of RR/HR/OR by calculation, tracheal intubation was not performed in a hospital. After reviewing the full texts, 16 studies (published between 1998 and 2015) were selected for inclusion (Fig. 1). No unpublished study in clinicaltrial.gov met the inclusion criteria.

### Study characteristics

A total of 204,303 subjects were included in this meta-analysis; 12,757 were assigned to the obese group, and 191,546 were assigned to the non-obese group. Five studies involving 100,974 patients were included in the analysis of the association between obesity and risk of difficult tracheal intubation [7, 23, 24]. Nine studies with a total of 112,388 patients were included in the obesity and difficult laryngoscopy group [7, 23–29]. Twelve studies with a total of 5678 patients were analysed the association between obesity and risk of Mallampati score ≥ 3 [7, 14, 23, 24, 26, 28–34]. There were five case-control studies (two in the USA [23, 28], one [27] in Germany, one in Denmark [35], and one in Ireland [32]) and 11 cohort studies (two in France [7, 24], two in Turkey [25, 31], one in Brazil [30], one in Israel [14], two in the USA [26], one in Greece [29] one in Italy [34] and one in the UK [33]). Two studies included parturients [25, 31], and 14 recruited non-parturients [7, 14, 23, 24, 26–30, 32–34]. Of the included studies, emergent tracheal intubation was used in two [23, 25] and non-emergent tracheal intubation in the other 14 [7, 14, 24, 26–34]. The sniffing position was employed in five studies [7, 14, 24, 26, 28] and the supine position in 11 [23, 25, 27, 29–34]. The definition of obesity as a BMI cut-off = 30 was applied in nine studies [7, 14, 23, 25, 28–32], whereas a BMI cut-off > 30 was used in seven studies [24, 26, 27, 33, 34]. The general characteristics of the published articles included in this meta-analysis are shown in Table 1.

**Table 1:** Characteristics of eligible trials

Trials	Country	Groups	Population(n) (Female/Male)	Studydesign	Subjects	Outcomes
Gonzalez 2008 [7]	France	obese(BMI≥30 kg/m2)	70	prospective cohort study	GA with endotracheal intubation	Rate of difficult intubation, difficult laryngoscopy and Mallampati score≥3
		non-obese(BMI<30 kg/m2)	61			
Mashour 2008 [8]	USA	obese (BMI≥40 kg/m2)	346	prospective cohort study	surgery under GA	Rate of difficult intubation and difficult laryngoscopy
		non-obese (BMI<40 kg/m2)	4827			
Lavi 2009[14]	Israel	obese(BMI≥30 kg/m2)	105	prospective cohort study	elective surgery with GA (exclusion emergent surgery)	Rate of Mallampati score≥3
		non-obese(BMI<30 kg/m2)	99			
Dargin 2013 [23]	USA	obese (BMI≥30 kg/m2)	338	case–control study	intubated in the ICUs, ED, and elsewhere in the hospital(excluded intubations performed in the operating room)	Rate of difficult laryngoscopy and Mallampati score≥3
		non-obese(BMI<30 kg/m2)	715			
Juvin 2003 [24]	France	obese(BMI≥35 kg/m2) non-obese(BMI<30 kg/m2)	129 134	prospective cohort study	Obese patients: laparoscopic gastroplasty; non-obese patients: inguinal hernia repair or laparoscopic cholecystectomy	Rate of difficult intubation, difficult laryngoscopy and Mallampati score≥3
Basaranoglu 2010[ 25]	Turkey	obese (BMI>30 kg/m2)	71	prospective cohort study	Consecutive women requiring GAfor emergency caesarean section	Rate of difficult laryngoscopy
		non-obese(BMI≤30 kg/m2)	168			
Ezri 2003 [26]	USA	obese(BMI>35 kg/m2)	200	prospective cohort study	obese patients undergoing laparoscopic weight reduction surgery (LapBand); non-obese patients underwent laparoscopic abdominal procedures	Rate of difficult laryngoscopy and Mallampati score≥3
		non-obese(BMI≤35 kg/m2)	1272			
Heinrich 2013 [27]	Germany	obese(BMI≥35 kg/m2)	6334	case-control study	undergoing general anesthesia for any type of surgical or diagnostic intervention	Rate of difficult laryngoscopy
		non-obese(BMI<35 kg/m2)	95556			
Lee 2015 [28]	USA	obese(BMI≥30 kg/m2)	160	case–control study	adult patients undergoing GA (exclusion emergency intubation)	Rate of difficult laryngoscopy and Mallampati score≥3
		non-obese(BMI<30 kg/m2)	184			
Voyagis 1998 [29]	Greece	obese (BMI≥30 kg/m2)	99	prospective cohort study	elective surgery under GA with endotracheal intubation( exclusion heart disease, emergency or obstetric surgery)	Rate of difficult laryngoscopy and Mallampati score≥3
		non-obese (BMI<40 kg/m2)	1734			
Magalhaes 2013 [30]	Brazil	obese(≥30 kg/m2)	43	prospective cohort study	undergoing GA for surgical procedures	Rate of Mallampati score≥3
		non-obese(<30 kg/m2)	45			
Turkay Aydogmus 2014 [31]	Turkey	obese(BMI>30 kg/m2)	20	prospective cohort study	pregnant women of ASA 1-2 scheduled for GA for cesarian operation	Rate of Mallampati score≥3
		non-obese(BMI≤30 kg/m2)	20			
Aslani 2012 [32]	Ireland	obese (BMI>30 kg/m2)	58	case control study	women referred to the anaesthetic clinic and relevant historic control groups	Rate of Mallampati score≥3
		non-obese(BMI<30 kg/m2)	34			
Combes 2005 [33]	United Kingdom	obese(BMI>35 kg/m2)	50	prospective cohort study	patients scheduled to undergo abdominal, orthopedic, or cardiac surgery GA(laryngeal mask)	Rate of Mallampati score≥3
		non-obese(BMI<30 kg/m2)	50			
Yildiz 2010 [34]	Italy	obese (BMI>40 kg/m2)	30	prospective cohort study	undergoing GA for gynecological surgery with use of the LMA CTrach	Rate of Mallampati score≥3
		non-obese (BMI<30 kg/m2)	30			
Lundstrøm 2009 [35]	Denmark	obese(≥35 kg/m2)	4704	case control study	all types of surgery (except for cardiothoracic surgery)	Rate of difficult intubation
		non-obese(<35 kg/m2)	86617			

*GA* general anesthesia, *NR* not reported, *ICU* intensive care unit, *ED* emergency department, *ASA* American Society of Anesthesiologists, *IDS score* the intubation difficulty scale score

### Quality assessment

The results of the quality assessment are presented in Table 2. The ROBINS I tool indicated an overall low to moderate risk of bias, which for the majority of studies originated from the selection of the reported results as well as from the presence of possible confounding factors.

**Table 2:** Risk of bias in non-randomised studies -of interventions (ROBINS-I) tool

Author; Year	Bias due to confounding	Bias in selection of participants into the study	Bias due to missing data	Bias in measurement of outcomes	Bias in selection of the reported result	Overall bias
Gonzalez 2008 [7]	Moderate	Low	Low	Low	Moderate	Moderate
Mashour, 2008 [8]	Moderate	Low	Low	Low	Low	Low
Lavi2009[14]	Moderate	Low	Low	Low	Moderate	Moderate
Dargin 2013 [23]	Moderate	Low	Moderate	Low	Moderate	Moderate
Juvin 2003 [24]	Moderate	Low	Low	Low	Moderate	Moderate
Basaranoglu 2010 [25]	Moderate	Low	Low	Low	Moderate	Moderate
Ezri 2003 [26]	Moderate	Low	Low	Low	Moderate	Moderate
Heinrich 2013 [27]	Moderate	Low	Moderate	Low	Moderate	Moderate
Lee 2014 [28]	Moderate	Low	Low	Low	Moderate	Moderate
Voyagis 1998 [29]	Moderate	Low	Low	Low	Moderate	Moderate
Magalhães 2013 [30]	Moderate	Low	Low	Low	Moderate	Moderate
Turkay Aydogmus 2014 [31]	Moderate	Low	Low	Low	Moderate	Moderate
Aslani2012[32]	Moderate	Low	Low	Low	Moderate	Moderate
Combes2005[33]	Serious	Low	Low	Low	Moderate	Moderate
Yildiz 2010 [34]	Serious	Low	Low	Low	Moderate	Moderate
Lundstrøm 2009 [35]	Moderate	Low	Low	Low	Low	Low

All parameters were assessed for their risk by using a scale that classifies them as low, moderate, serious or critical

### Association between obesity and rate of difficult tracheal intubation

**Fig. 2:**
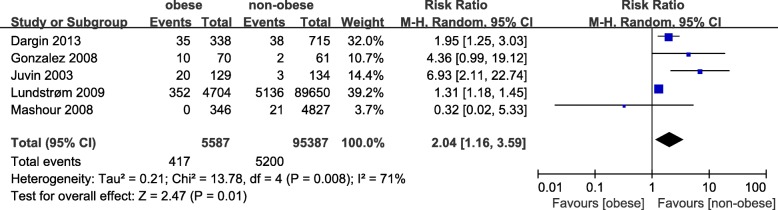
The result is the pooled estimate of the 5 included studies by random effect model. RR: rate ratios. There was a statistically significant association pooled (RR = 2.04, 95% CI: 1.16–3.59, *p* = 0.01; I2 = 71%, *p* = 0.008, Power = 1.0) between obesity and risk of DI

**Fig. 3:**
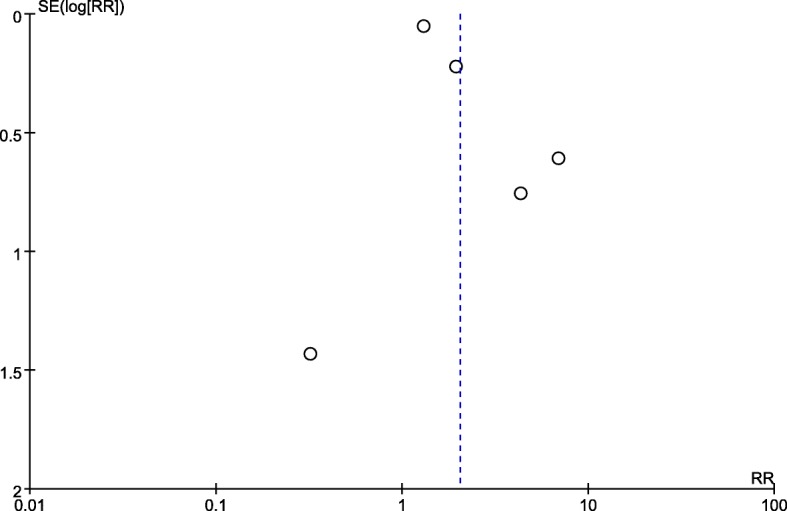
No obvious asymmetry was detected in the funnel plots

Five studies with a total of 100,974 patients were included in this analysis [7, 8,23, 24, 35]. There was a significant association (pooled RR = 2.04, 95% CI: 1.16–3.59, *p =* 0.01; I^2^ = 71%, *p =* 0.008, Power = 1.0) between obesity and risk of DI (Fig. 2). Subgroup analysis of case-control studies showed that obesity was associated with an increased risk of DI (pooled RR = 1.50, 95% CI: 1.03–2.18, *p =* 0.03; I^2^ = 67%, *p =* 0.08). Similarly, obesity was associated with an increased risk of DI in both the sniffing (pooled RR = 5.77, 95% CI: 2.29–14.58, *p =* 0.0002; I^2^ = 0%, *p =* 0.63) and supine (pooled RR = 1.30, 95% CI: 1.17–1.44, *p =* 0.04; I^2^ = 64%, *p =* 0.02) positions. However, subgroup analysis of cohort studies revealed no trend of obese patients having a higher risk of DI compared with non-obese patients (RR = 3.41, 95% CI: 0.88–13.23, *p = 0.08*; I^2^ = 50, *p =* 0.14) (Table 3). We sequentially removed each study and then reanalysed the remaining dataset; removal of the studies defined obesity as a BMI cut-off > 30 [24], reducing the heterogeneity without significantly affecting the RR (RR = 2.12, 95% CI: 1.30–3.47, *p = 0.003*; I^2^ = 6, *p =* 0.30). Nonetheless, there was no significant difference in the estimates after we excluded studies with emergency tracheal intubation (RR = 2.31, 95% CI: 0.76–6.99, *p = 0.14*; I^2^ = 73, *p =* 0.01). No obvious asymmetry was detected in funnel plots (Fig. 3).

**Table 3:** Subgroup analysis of the outcomes

Study group	Outcomes									
	Number of studies (n)	Number of participants (n)	test of association			Test of heterogeneity				
			RR	95%CI	*p* value	Model	*p* value	I^2^	I^2^ test for subgroup differences (%)	*p* for the interaction between subgroup and treatment
difficult intubation										
*Study design*										
Cohort	3	5567	3.41	0.88–13.25	0.08	RE	0.14	50	23	0.25
Case-control	2	95,407	1.50	1.03–2.18	0.03	RE	0.08	67		
*Position*										
Sniffing	2	394	5.77	2.29–14.58	0.0002	RE	0.63	0	89.8	0.002
Supine	3	100,570	1.30	1.17–1.44	< 0.00001	RE	0.59	0		
difficult laryngoscopy										
*Study design*										
Cohort	6	9111	1.85	1.31–2.63	0.0005	RE	0.08	49	67	0.08
Case-control	3	103,277	1.34	1.22–1.48	< 0.00001	RE	0.87	0		
*Position*										
Sniffing	4	2210	2.0	0.97–4.15	0.06	RE	0.03	67	0	0.42
Supine	5	110,178	1.47	1.23–1.76	< 0.0001	RE	0.22	30		
Mallamp-ati ≥ 3										
*Study design*										
Cohort	9	4189	1.9	1.12–3.21	0.02	RE	< 0.00001	85	0	0.6
Case-control	3	1489	1.62	1.27–2.08	0.0001	RE	0.61	0		

*CI* confidence interval, *RE* random-effect model, *RR* risk ratio, *I*^*2*^ a test for heterogeneity, I^2^ > 50% indicates substantial heterogeneity

### Association between obesity and the rate of difficult laryngoscopy

**Fig. 4:**
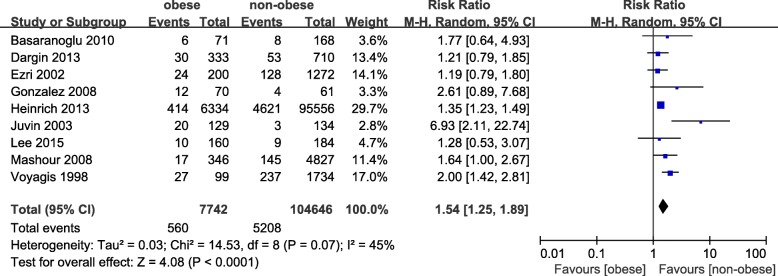
The result is the pooled estimate of the 9 included studies by random effect model. RR: rate ratios. There was a statistically significant association (pooled RR = 1.54, 95% CI: 1.25–1.89, *p* < 0.0001; I2 = 45%, *p* = 0.07, Power = 1.0) between obesity and risk of difficult laryngoscopy

**Fig. 5:**
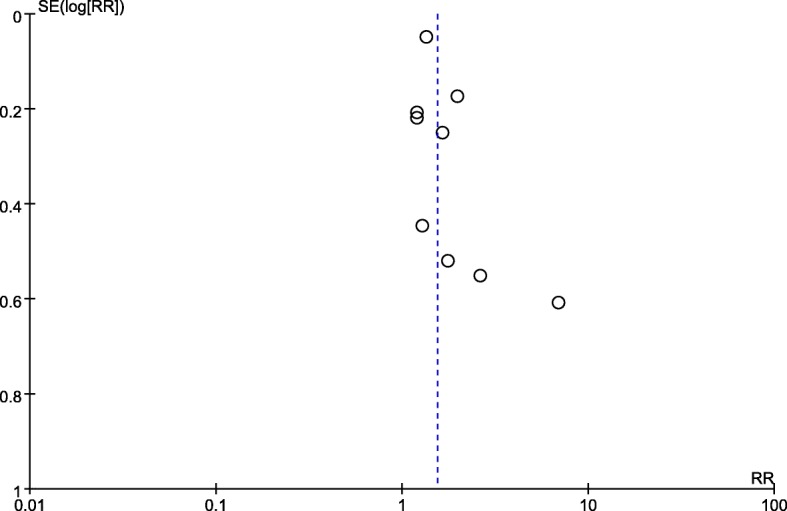
No obvious asymmetry was detected in the funnel plots

Nine studies including 112,388 patients were evaluated [7, 23–29]. There was a statistically significant association (pooled RR = 1.54, 95% CI: 1.25–1.89, *p <* 0.0001; I^2^ = 45%, *p =* 0.07, Power = 1.0) between obesity and the risk of difficult laryngoscopy (Fig. 4). The results of subgroup analyses are presented in Table 3. Obesity was associated with an increased risk of difficult laryngoscopy in both cohort studies (pooled RR = 1.85, 95% CI: 1.31–2.63, *p =* 0.0005; I^2^ = 49%, *p =* 0.08) and in case–control studies (pooled RR = 1.34, 95% CI: 1.22–1.48, *p <* 0.00001; I^2^ = 0%, *p =* 0.87). Similarly, obesity was associated with an increased risk of difficult laryngoscopy in the supine position (pooled RR = 1.47, 95% CI: 1.23–1.76, *p < 0.0001*; I^2^ = 45%, *p =* 0.07). However, subgroup analysis showed that compared with non-obesity, there was no association with the risk of difficult laryngoscopy in obese patients in the sniffing position (pooled RR = 2.00, 95% CI: 0.97–4.15, *p = 0.06*; I^2^ = 67%, *p =* 0.03). We sequentially removed each study as well as studies with parturients and emergency tracheal intubation and then reanalysed the remaining dataset; there were no major changes in the direction or magnitude of the statistical findings. However, removal of studies defining obesity as a BMI cut-off > 30 [24, 26, 27] reduced heterogeneity without significantly affecting the RR (RR = 1.64, 95% CI: 1.26–2.14, *p = 0.002*; I^2^ = 9, *p =* 0.36). No evidence of publication bias was evident by visual inspection of a funnel plot (Fig. 5).

### Association between obesity and Mallampati score ≥ 3

**Fig. 6:**
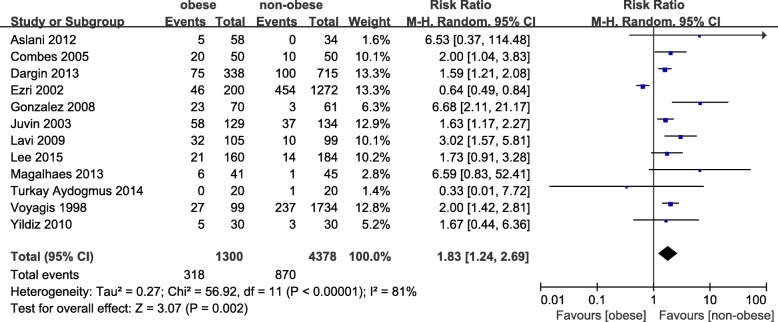
The result is the pooled estimate of the 12 included studies by random effect model. RR: rate ratios. There was a statistically significant association (pooled RR = 1.83, 95% CI: 1.24–2.69, *p* = 0.002; I^2^ = 81%, *p* < 0.00001, Power = 0.93) between obesity and risk of Mallampati score > 3

**Fig. 7:**
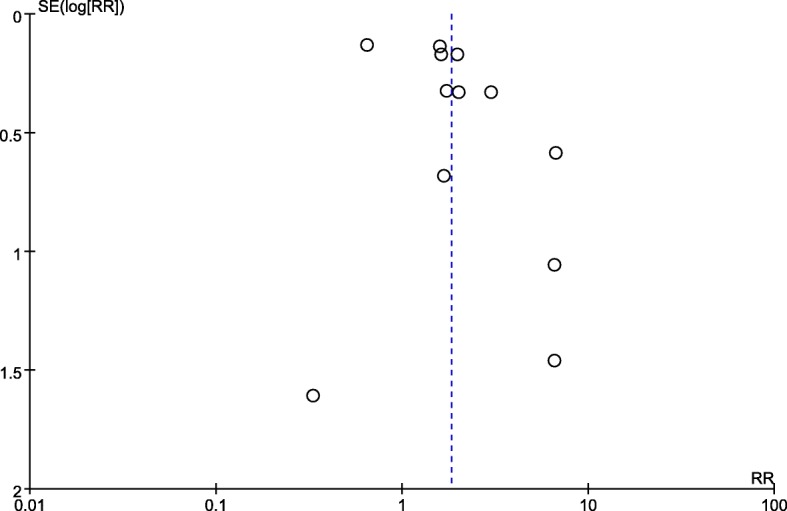
No obvious asymmetry was detected in the funnel plots

Twelve studies with a total of 5678 patients were analysed [7, 14, 23, 24, 26, 28–34]. There was a significant association (pooled RR = 1.83, 95% CI: 1.24–2.69, *p =* 0.002; I^2^ = 81%, *p <* 0.00001, Power = 0.93) between obesity and a Mallampati score ≥ 3 (Fig. 6). The results of subgroup analyses are presented in Table 3. Obesity was associated with an increased rate of a Mallampati score ≥ 3 in both cohort (pooled RR = 1.90, 95% CI: 1.12–3.21, *p =* 0.02; I^2^ = 85%, *p <* 0.00001) and case-control (pooled RR = 1.62, 95% CI: 1.27–2.08, *p =* 0.0001; I^2^ = 0%, *p = 0.61*) studies. We sequentially removed each study and those that included emergency tracheal intubation or parturients and then reanalysed the remaining dataset. Although there were no major changes in the direction or magnitude of the statistical findings, removal of studies defining obesity as a BMI cut-off > 30 decreased heterogeneity without significantly affecting the RR (RR = 2.14, 95% CI: 1.55–2.96, *p < 0.00001*; I^2^ = 41, *p =* 0.10). No evidence of publication bias was evident by visual inspection of the funnel plot (Fig. 7).

## Discussion

To the best of our knowledge, this is the first meta-analysis focusing on evaluating the association between obesity with risk of DI in recent decades. The findings revealed a significant association between obesity and the rate of difficult tracheal intubation, difficult laryngoscopy and Mallampati score ≥ 3. However, subgroup analysis showed no trend of a higher association of obesity with a risk of DI compared with non-obesity in cohort studies and no association with an increased risk of difficult laryngoscopy in the sniffing position. In addition, no trend of obese patients having a higher association with DI risk compared with non-obese patients when removing emergency tracheal intubation was found in sensitivity analyses.

There are conflicting reports regarding the correlation of BMI with DI [7, 8, 35]. Two prospective studies found no correlation between BMI and DI [7, 8], whereas a retrospective study of large samples concluded that the correlation was weak but statistically significant [35]. These discrepancies may be explained by methodological differences and varying study designs. As a consequence, large-sample, sufficient-power and high-level evidence studies are essential. The powers of our outcomes were 1.00, 1.00 and 0.93, and we believe that these were sufficient because most researchers assess power using 0.80 as a standard for adequacy. Furthermore, to control for the Type I error rate, we used Bonferroni adjustment to control the significance criterion. As the overall qualities of the studies were satisfactory, we consider our results to be convincing.

Importantly, we chose difficult tracheal intubation as our primary outcome because clinicians want to know whether this procedure is more difficult in obese than in non-obese patients. However, difficult tracheal intubation has many influencing factors. Indeed, DI represents a complex interaction between patient factors, the clinical setting, and the skill of the practitioner. As a consequence, we chose the rates of difficult laryngoscopy and a Mallampati score ≥ 3 to be our secondary outcomes. The direct reason for difficult tracheal intubation by DL is the difficult laryngoscopy procedure, and the extent of laryngoscopy is an important prediction method for intubation that is widely used in clinical practice. In addition, the Mallampati score is a preoperative assessment widely applied due to its better alignment with Cormack-Lehane grades [36]. As a result, we believe that the three outcomes complement each other and are indispensable for evaluating difficult intubation.

It is worth mentioning that for the outcome of DI, the CIs obtained varied widely, and the heterogeneity was high. To identify sources of heterogeneity, we utilized analysis of prespecified subgroups and sensitivity analyses. However, subgroup analysis showed no significant association of obesity with DI risk compared with non-obesity in cohort studies. In obese patients, emergency tracheal intubation can be particularly challenging because of the increased risk of impaired respiratory mechanics [37, 38], and poor tolerance of apnea [39–41]. Therefore, we conducted sensitivity analyses by removing emergency tracheal intubation and found no significant differences in the estimates of elective tracheal intubation (RR = 2.31, 95% CI: 0.76–6.99, *p = 0.14*; I^2^ = 73, *p =* 0.01). Moreover, heterogeneity was reduced without significantly affecting the RR by removal of studies defining obesity as a BMI cut-off > 30 (RR = 2.12, 95% CI: 1.30–3.47, *p = 0.003*; I^2^ = 6, *p =* 0.30) [8, 24, 35], suggesting that this result was unstable.

The sniffing position has been commonly advocated as the standard head position for DL. In this position, the neck must be flexed on the chest, typically by elevating the head with a cushion under the occiput and extending the head on the atlanto-occipital joint [42, 43]. Regardless, the anatomic explanation of the advantage of the sniffing position has been called into question [44–46]. In subgroup analysis, obesity was not associated with an increased risk of difficult laryngoscopy in the sniffing position compared with non-obesity. This was a believable result, as it is based on a large sample of high-quality research, and it confirms the effect of the sniffing position in improving the laryngeal view in obese patients.

According to the Cochrane Collaboration common scheme for bias and the ROBINS-I tool, the studies demonstrated low/moderate risk of bias. For the majority of the studies, this bias originated from the selection of the reported results as well as from the presence of possible confounding factors. These studies had higher levels of evidence.

There were some limitations to this meta-analysis. First, methodologic limitations with regard to the studies and statistical heterogeneities among the studies were significant. Some biases were unavoidable. For example, it was not possible to blind either anaesthesiologists or patients regarding non-obese or obese patients. Thus, it is accepted that observational studies were essentially included. Second, we only explored difficult tracheal intubation by direct laryngoscopy and not by difficult airway, therefore lacking facemask data. As the risk factors for difficult mask ventilation and DI are quite different [47], future analyses should explore the association between BMI and difficult airway.

## Conclusions

Current meta-analysis indicated that obesity was associated with an increased risk of DI, difficult laryngoscopy and a Mallampati score ≥ 3 in adults patients undergoing general surgical procedures. However, there was no association between obesity and risk of DI compared with non-obesity in cohort studies and elective tracheal intubation and no association between an increased risk of difficult laryngoscopy in the sniffing position. Nonetheless, high heterogeneity among the studies included in this analysis limits the generalizability of our findings. Future analyses should explore the association of BMI with difficult airway.

## Abbreviations

ASA: American Society of Anesthesiology
BMI: Body mass index
CI: Confidence interval
DI: Difficult intubation
DL: Direct laryngoscope
RR: Risk ratios
